# Creation of a federated database of blood proteins: a powerful new tool for finding and characterizing biomarkers in serum

**DOI:** 10.1186/1559-0275-11-3

**Published:** 2014-01-29

**Authors:** John Marshall, Peter Bowden, Jean Claude Schmit, Fay Betsou

**Affiliations:** 1 Department of Chemistry and Biology, Ryerson University, Toronto, Canada; 2 Luxembourg Center for Clinical Proteomics, CRP SANTE, 1A-B, rue Thomas Edison L-1445 Strassen, Luxembourg, Luxembourg; 3 Integrated Biobank of Luxembourg, 6, rue Nicolas Ernest Barblé, Luxembourg L-1210, Luxembourg

## Abstract

Protein biomarkers offer major benefits for diagnosis and monitoring of disease processes. Recent advances in protein mass spectrometry make it feasible to use this very sensitive technology to detect and quantify proteins in blood. To explore the potential of blood biomarkers, we conducted a thorough review to evaluate the reliability of data in the literature and to determine the spectrum of proteins reported to exist in blood with a goal of creating a Federated Database of Blood Proteins (FDBP). A unique feature of our approach is the use of a SQL database for all of the peptide data; the power of the SQL database combined with standard informatic algorithms such as BLAST and the statistical analysis system (SAS) allowed the rapid annotation and analysis of the database without the need to create special programs to manage the data. Our mathematical analysis and review shows that in addition to the usual secreted proteins found in blood, there are many reports of intracellular proteins and good agreement on transcription factors, DNA remodelling factors in addition to cellular receptors and their signal transduction enzymes. Overall, we have catalogued about 12,130 proteins identified by at least one unique peptide, and of these 3858 have 3 or more peptide correlations. The FDBP with annotations should facilitate testing blood for specific disease biomarkers.

## Introduction

Most human diseases involve the changes in the expression of normal proteins, or the creation of abnormal proteins, that disrupt physiology; such protein changes can arise from mutations, viruses, chemicals, radiation, free radicals, ischemia, disease or other the sources. In many instances these proteins may appear in blood thus providing an easily accessible biomarker that may give insight into the disease process.

Proteins that are specifically expressed in certain tissue types or cells may be useful markers of disease. Prostate specific antigen (PSA) is not selective for malignant disease but is a tissue-specific kalikrein protease that indicates benign and cancerous proliferation of prostate tissue and cells [1]. Transcription factors, DNA binding and RNA binding zinc finger proteins, chromatin remodeling proteins, cellular receptors or agonists, and cell signaling proteins including oncogenes that may play a role in cell and tissue differentiation [2], were first reported from the tryptic peptides of blood fluid after partition chromatography [3,4]. The presence of so many nuclear and cellular factors was unexpected given previous views on the nature of plasma proteins [5,6]. Now it appears that the nucleic acid and protein binding factors that play a regulatory or signal function in specific cell types [2] may frequently be detected in blood and have clinical diagnostic/prognostic or even therapeutic value [7-9].

The proteins and endogenous peptides of human plasma may be randomly and independently sampled, identified by liquid chromatography and tandem mass spectrometry, followed by log transformation and comparative statistical analysis by ANOVA [10-12]; with the current generation of mass spectrometers, these processes are relatively simple and accessible to many laboratories. However, to facilitate routine use in biomarker studies, we have created a federated database of all proteins and peptides reported in blood. Here we provide for the first time the specific identity of the set of cellular regulatory proteins detected with high confidence and good agreement internationally from human serum/plasma by liquid chromatography and tandem mass spectrometry that show great promise as potential biomarkers and therapeutic targets. The good agreement on peptides from the same cellular proteins by different research groups, together with the capacity of mass spectrometry to compare blood samples at the level of the parent peptide and fragment ion intensity using ANOVA, indicates that conditions for the detailed analysis of blood fluids between diseases and physiological states may now exist [10-12].

Although several organizations and individuals have published large data sets of proteins in blood, it remains to provide an SQL database by a systematic compilation and analysis of publically available data sets. This mathematical review of published data evaluates the reliability of the data in 11 published reports [13-23] and includes a federated database of all of the proteins and peptides detected in blood. The new SQL based, federated database of blood proteins (FDBP) available *in toto* with this publication will provide a good starting point for groups wishing to evaluate various peptides in blood as biomarkers of disease.

## Construction and analysis of the federated database of blood proteins

### Federated protein sequence library

A federated database of protein sequences was assembled by downloading protein sequences from RefSeq, Ensembl, SwissProt, Trembl or Uniprot that contain sequences from many sources to yield a non-redundant set of proteins, protein fragments, splice variants and alleles that contains over 193,000 possible protein sequences representing different genes, isoforms, transcript lengths, splice variants, alleles, and recorded nucleic sequences.

### Meta database approach

A separate computer program was written to parse each of the publicly available serum and plasma proteomic data [13-23]. The data of Zhu et al [20] is a reanalysis of the raw data of Marshall et al [3]. The data of Bowden et al is the analysis of the raw data of the HuPO PPP consortium downloaded from TRANCHE website and calculated by X!TANDEM [23]. The results were reported in different formats with accession numbers and FASTA protein sequences obtained from RefSeq, Swiss, protein, ENSEMBL, Uniprot IPI or other predicted protein sequences. To facilitate the easy analysis, the peptides and proteins from the independently published reports of human serum/plasma proteins were then transferred to a single SQL database. It has been previously suggested that it is challenging to derive high confidence identifications of human serum/plasma proteins [4] and that most proteomic identifications are false positive results using the “empirical model” based on putatively pure protein standards verses a decoy library [24]. However, statistical confidence in protein identification certainly increases through replication and independent identification of the same proteins [25,26]. Moreover the so called FDR used in proteomics, based on the empirical model, has been shown to disagree with classical statistical analyses by many orders of magnitude and to incorrectly reject well known blood proteins, including albumin, resulting is a large type II error (false negative) of protein identification [10,27]. Comparing the goodness-of-fit scores of authentic MS/MS spectra versus random or noise spectra [27,28] shows that the score distributions of real spectra correlations can be easily separated from false positive results [27,28] so long as the data are collected with a high signal to noise ratio [10-12].

Confidence is known to increase with the number of peptides identified from each protein [25,26] and so the peptide to protein distribution of a data set is a simple means to infer the statistical reliability of the data [10-12]. Previously, control experiments using random libraries and random or noise spectra showed that roughly 88% of false positive proteins were identified by 1 peptide, about 11% of false positive hits show 2 peptides, and about 1% of false positive hits show 3 or more peptides: Experiments with random libraries of amino acid sequences, decoy libraries and random spectra all agree that proteins observed with at least 3 peptides show a false positive rate of less than 1% [27,28] and a low false positive rate (type I error) [25,27]. LC-ESI-MS/MS data sets that have been recorded with a high signal to noise ratio in replicated experiments show up to tens or hundreds of peptide detections per protein and thus a low probability of false positive results. In contrast, the peptide to protein distribution of noise spectra was not different from random spectra [27,28]. An emphasis on replication, and good agreement between independent experiments, is a pragmatic method to provide confidence in sensitive ion trap data.

### Analysis of the data sets

The database of HuPO plasma proteome results [23] and all previously published blood results [29] were obtained as previously described [20]. The HuPO consortium raw data was obtained from the TRANCHE website and analyzed with X!TANDEM [29,30]. The resulting proteins were detected from independent experiments using different instruments and/or correlation algorithms including X!TANDEM [19], MASCOT, OMSSA or others [18] and SEQUEST [31,32]. The advantages and disadvantages of these approaches have previously been considered and the statistical reliability of the data set has previously been estimated [29]. The protein and peptide sequences reported, or where required the accession numbers, were used to map all of the previous blood fluid results to the federated protein library to create the FDBP.

The federated sum of the peptide and protein sequences reported, and the number of observations, were calculated using Structured Query Language (SQL). The SQL server database system was installed on a personal computer (PC) using the Windows server operating system to create the FDBP: The redundant and distinct protein sequences were determined using SQL [29]. The resulting FDBP was subsequently analyzed on a 64 bit PC with SQL Access as previously described [23].

The BLAST (Basic Local Alignment of Sequence Tool) algorithm [33] provides a standardized way to search for homologous regions on proteins and quantify the level of similarity within a set of proteins; BLAST was downloaded from NCBI and installed on a PC [33]. A subset of blood protein results were organized into a BLAST matrix for the purpose of comparing BLAST to the automatic features of SQL. The results of the BLAST analysis for each sequence were also captured in the SQL database alongside the corresponding proteins. The top scoring BLASTp alignment for every query protein was considered a “match” if the identity was greater than 75% of the full length and if the alignment contained a perfect match string of at least 20 amino acids [29].

The SQL database of the peptides and proteins mapped to the federated protein library was explored using SAS to generate the graphs provided [23]. The data were graphed in SAS JMP after sorting the parameter(s) of interest in descending order using the Tables - Sort menu. The resulting data was plotted versus increasing protein number using the graphic features inherent to the SAS. The peptide to protein distributions of the blood data compared to that of randomized libraries [25] or randomized spectra or noise indicate that the proteins detected by three or more different peptides show a low expectation of false positive results ≤ 1% FDR [10,23,27-29]. The graphs were exported from SAS as rich text format files. The data were analyzed on a 64 bit PC with SAS JMP as previously described [23]. After comparing BLAST versus SQL we imported the peptides, proteins, and ion characteristics including intensity values from Liu et al 2007 and included these in the SQL database only for summary of the FBPD.

The peptide and protein sequences were used as queries to obtain the protein descriptions from RefSeq, Ensembl, Swiss Prot and other protein libraries that were concatenated with semicolons. Similarly BLAST was used to obtain the molecular identity or molecular functions, biological processes and sub cellular localization of the proteins found in human blood where gene ontology terms (GO) and gene symbols were available. The peptide and protein sequences along with the accession numbers, GO terms and the results of bio-informatic calculations from the GO and BLAST analysis are organized and stored together.

The cellular factors of specific interest from the FDBP are made available in the Additional files. The cellular proteins of blood fluids listed in the FDBP are presented in a graphical form using the Search Tool for the Retrieval of Interacting Genes/Proteins (STRING) [34] that automatically generated a list of gene symbols from the protein descriptions provided in the FDBP. However, more complete information can be found in the Additional files.

## Results

### Comparison of SQL versus BLAST analysis

We compared the BLAST analysis of amino acid sequences versus SQL analysis at the level of peptides and proteins on a large subset of the data to compare the methods and characterize the distributions of blood protein data. BLAST analysis [33] of a set of 44,019 reported proteins at a standard of 75% full length and 20 contiguous amino acids, compressed these to a set of 17,506 protein types. Among the proteins, 14,224 had no close homologues in the reference library of protein sequences; the remaining proteins occurred at least twice in the FDBP. After compression by BLAST a set of 7,707 proteins types were detected by at least three peptides. Based on the BLAST analysis the available annotation such as descriptions, GO data and accession numbers could be connected with the appropriate database entry.

#### Homology expectation value

**Figure 1:**
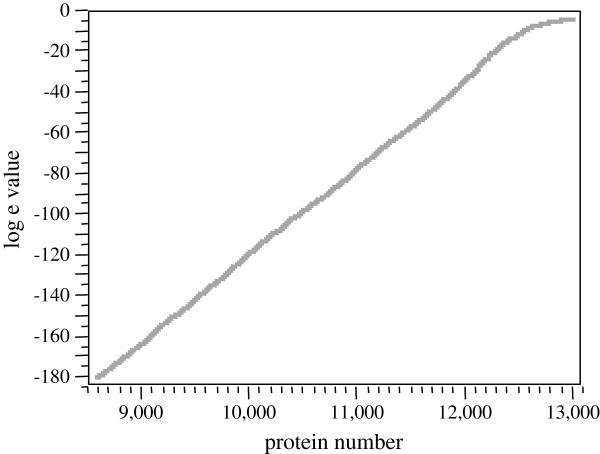
**The probability of homology between a subset of 27,254 distinct blood proteins as determined by the BLAST algorithm.** Note that about eight thousands proteins matches showed probability values less that E-180 (machine 0) and so are not shown.

The chance that the homology observed between proteins was just a random coincidence is the expectation value (e). The expectation value of the homology observed between the reported serum/plasma proteins was determined by the BLAST algorithm. Some 13,010 proteins were found to have significant homology in the FDBP by BLAST. The distribution of BLASTp significance values were captured in the SQL database and plotted in SAS (Figure 1). Note that ~ eight thousand protein matches showed probability values less than E-180 (machine 0) and so are not shown. BLAST reduction compressed the 13,010 proteins into about 3262 protein types (Figure 1).

#### Sequence gap analysis

**Figure 2:**
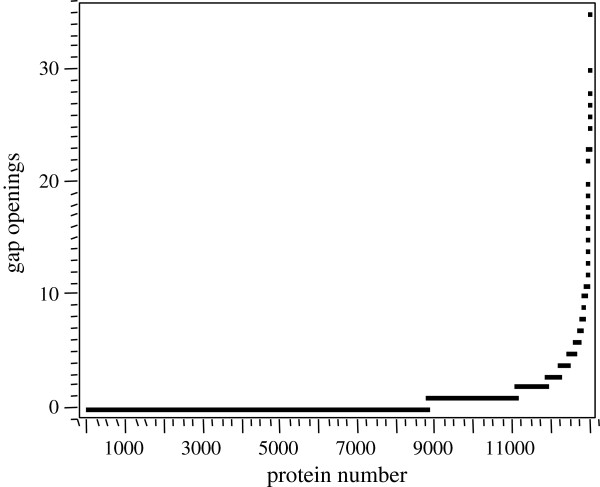
**The distribution of gap openings in homologous proteins as calculated by BLAST.** Note that almost 9000 protein matches showed perfect alignments with no gaps in the matched amino acid sequences. In contrast, a small subset of about one thousand proteins showed three or more gaps in the matched sequence.

Proteins that have homology may show strings of sequence with perfect alignment, interspersed by breaks or openings where the sequences are not similar or missing. The distribution of gap openings in homologous proteins was calculated by BLAST (Figure 2). Note that almost 9000 protein matches showed perfect alignments with no gaps in the matched amino acid sequence. In contrast, a small subset of about one thousand proteins showed three or more gaps in the matched sequence. In a small number of proteins more than 30 gaps were observed along the length of the homologous proteins (Figure 2).

#### Protein alignment length

**Figure 3:**
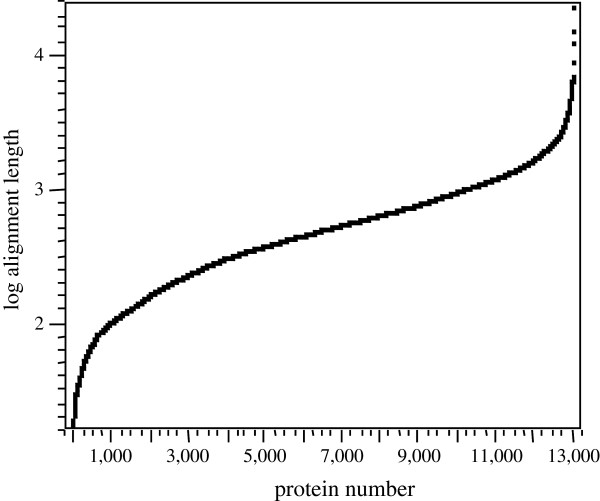
**The distribution of Log**_**10**_**protein match alignment lengths.** Note that almost 13,000 protein matches showed protein alignments of greater than 100 contiguous amino acids. Typically a contiguous stretch of 20 amino acids is considered sufficient evidence to indicate a potential structural relationship between proteins.

The distribution of Log_10_ protein match alignment lengths was calculated by BLAST (Figure 3). Almost 13,000 protein matches showed protein alignments of greater than 100 contiguous amino acids. Typically, a contiguous stretch of 20 amino acids is considered sufficient evidence to indicate a structural relationship between proteins.

#### Protein mis-matches

**Figure 4:**
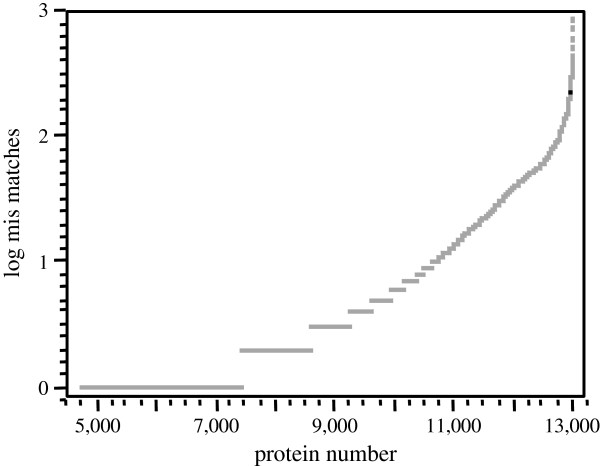
**The plot of log**_**10 **_**mis matches to protein match number.** Note that more than seven thousand proteins had few or no mis matches along the protein length. In contrast about four thousand proteins showed between 10 and one thousand mis-matches along the matched protein length.

Sometimes there are strings of sequence that in general show homology but have short regions where the sequence is not identical. The plot of log mismatches to proteins was calculated by BLAST (Figure 4). More than four thousand proteins had zero mismatches along the protein length. In contrast, about two thousand proteins showed at least ten, to as high as one thousand mis-matches along the protein length.

#### BLAST percent identity

**Figure 5:**
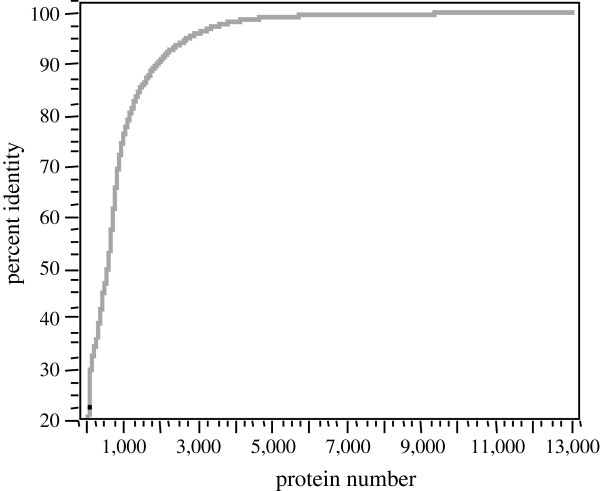
**The plot of percentage identity between protein matches.** Note that some twelve thousand protein matches show at least 70% identity over the full length of the query sequence that typically indicates a strong structural relationship between the protein sequences.

The plot of percentage identity between protein matches was calculated by BLAST (Figure 5). Note that some twelve thousand protein matches show at least 75% identity over the full length of the query sequence that typically indicates a clear structural relationship between the protein sequences.

#### SQL analysis

SQL analysis is based on the peptide or protein sequences. Liquid chromatography, coupled to electrospray ionization with tandem mass spectrometry can identify thousands of protein types, but there may be ambiguity in the results when there is a low level of peptide coverage and the peptides are shared by more than one protein. A total of 75,432 peptides produced a list of 57,784 peptides after the removal of duplicates using the distinct function of SQL. However, some of these peptides represented smaller pieces of other peptides and removal of these subsets of peptides gave 50,452 unique peptide sequences.

#### Redundant proteins by SQL

**Figure 6:**
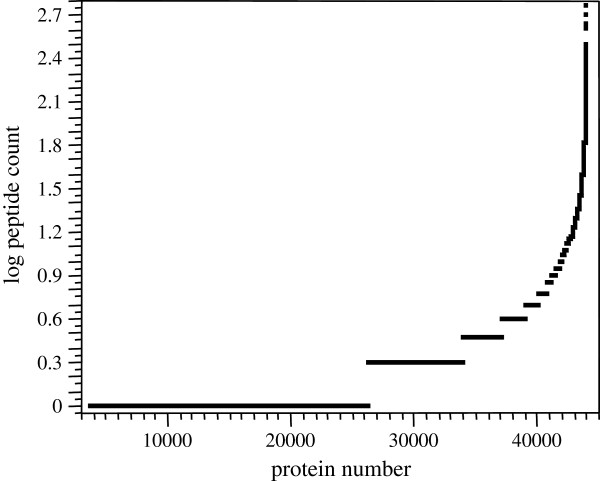
**The log**_**10 **_**peptide to protein distribution of the human blood proteins.** A set of published human blood data were parsed into SQL and the distributions of the data derived and graphed in SAS JMP.

Analysis of these raw data returned a total of 44,019 proteins of which 10,056 had three peptides or more; however, many proteins had identical sequences, but different protein names or accession numbers. The redundant peptide to protein count for the raw data showed just over half the proteins from each group separately had only one peptide reported but that a set of about ten thousand had three or more peptides including some proteins with up to 500 redundant identification (Figure 6). Hence the redundant peptide to protein distribution was observed to be markedly different from that of random expectation that should show a large proportion of protein with single peptides and almost no proteins with high numbers peptides.

#### Distinct proteins by SQL

**Figure 7:**
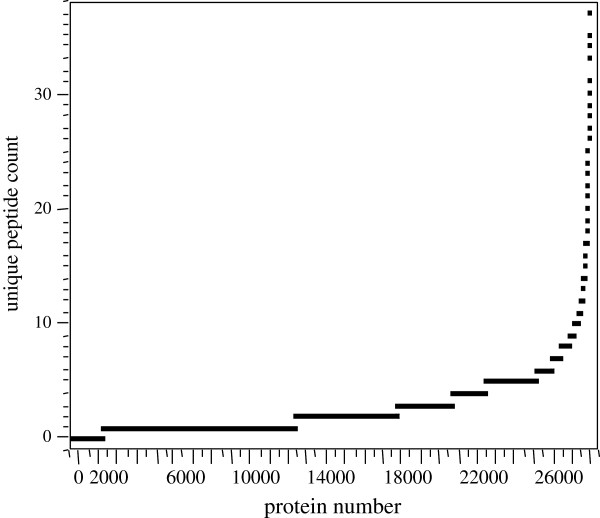
**The plot of distinct peptide count versus distinct protein number.** Note that about 12,000 proteins were only detected by 1 peptide. In contrast, a total of 10,138 distinct protein sequences were correlated by 3 or more different peptide sequences.

Removal of the duplicate proteins gave 27,254 distinct proteins that differed by at least 1 amino acid. After removing the proteins that were perfect subsets of other sequences, a total 10,138 unique protein sequences were identified by 3 or more distinct peptide sequences (Figure 7). Based on the distinct peptide distribution, we concluded that SQL showed similar trends, but that BLAST reduction may collapse some proteins together that are truly distinct but have some similar sequence.

### Unique or characteristic peptide sequence summary by SQL

There are many methods that can be used to estimate the vital statistics of the blood proteome, and perhaps the most conservative method would be to consider only proteins identified by at least one peptide that is unique to that protein and not characteristic of any other protein. An analysis of all the data reveals a set of 91,373 peptides from published studies on human serum/plasma of which 12,130 proteins that were detected by at least one unique peptide not shared with other proteins and of these 3858 had a total of at least three peptide identifications, conferring near certain molecular identity.

### The types of proteins detected in blood/serum

#### Broad spectrum of proteins detected in blood/serum

A major goal of this review was to determine the spectrum of proteins present in plasma/serum. A large number of proteins/peptides detectable in blood will make feasible the search for many biomarkers of disease processes. In addition to the usual proteins expected in the blood e.g., albumin, haemoglobin, gamma globulin, fibrinogen, ferritin, etc, many intracellular proteins from different tissues were found in the FDBPs. We transferred the annotations found in various databases to our FDBP and then used the SQL database to analyze the various classes of proteins. All cellular locations were observed in the dataset including the nucleus, integral membrane, cytosol and extracellular matrix (Table 1). The most common molecular functions were protein binding, DNA binding, “unknown”, DNA binding, Ca^++^ binding, Zn^++^ ion binding and receptor activity (Table 2). The most common biological processes observed were DNA-dependent transcription regulation, proteolysis, transport, signal transduction and metabolic processes (Table 3). The following sections give a summary the major classes of proteins found in blood.

**Table 1:** The distribution of cell location in the blood protein SQLdatabase

**Cellular location**	**Count**	**Frequency**
Total	22926	1
Nucleus,	2958	0.12902
Membrane, integral to membrane,	1330	0.05801
Cytoplasm,	810	0.03533
		
Extracellular region,	624	0.02722
Integral to membrane,	531	0.02316
		
Intracellular,	447	0.0195
Nucleus, cytoplasm,	414	0.01806
Intracellular, nucleus,	403	0.01758
Extracellular space,	363	0.01583
Membrane,	298	0.013
Mitochondrion,	269	0.01173
Plasma membrane, integral to membrane,	265	0.01156
Extracellular region, extracellular space,	264	0.01152
Cellular_component,	203	0.00885
Ubiquitin ligase complex,	200	0.00872
Ubiquitin ligase complex,	191	0.00833
Extracellular region, proteinaceous extracellular matrix,	179	0.00799
Nucleus, cytoplasm,	142	0.00619
Nucleus, nucleus,	131	0.00571
Plasma membrane, integral to plasma membrane,	129	0.00563
Integral to plasma membrane, membrane,	125	0.00545
Plasma membrane, integral to plasma membrane,	103	0.00449
Cytoskeleton,	95	0.00414
Proteinaceous extracellular matrix,	93	0.00406
		
		
Endoplasmic reticulum, endoplasmic reticulum membrane, membrane, integral membrane,	80	0.00349
Nucleosome, nucleus, chromosome,	78	0.0034
Intracellular, ribosome,	74	0.00323
Plasma membrane,	73	0.00318
Intracellular, cytoplasm,	72	0.00314
Lysosome,	72	0.00314
Intracellular, nucleus, cytoplasm,	71	0.0031
Actin cytoskeleton,	70	0.00305
Endoplasmic reticulum,	69	0.00301
Cytoplasm, cytoskeleton,	68	0.00297
Plasma membrane, integral to membrane,	66	0.00288
Cytosol,	65	0.00284
Intracellular, nucleus,	64	0.00279
Membrane fraction, integral to plasma membrane,	64	0.00279
Ubiquitin ligase complex, nucleus,	63	0.00275
Membrane fraction, integral to plasma membrane, membrane,	60	0.00262

For more complete information see Additional files.

**Table 2:** The distribution of molecular functions in the blood protein SQL database

**Molecular function**	**Count**	**Frequency**
Total	24031	1
Protein binding,	1373	0.05713
DNA binding,	348	0.01448
Binding,	340	0.01415
Calcium ion binding,	226	0.0094
Transcription factor activity,	225	0.00936
Structural molecule activity,	196	0.00816
Receptor activity,	193	0.00803
Calcium ion binding, protein binding,	185	0.0077
DNA binding, zinc ion binding,	179	0.00745
DNA binding, zinc ion binding, metal ion binding,	159	0.00662
Structural constituent of ribosome,	149	0.0062
Nucleic acid binding, zinc ion binding,	134	0.00558
Protein binding, zinc ion binding, metal ion binding,	125	0.0052
Catalytic activity,	109	0.00454
Nucleic acid binding,	106	0.00441
Calcium ion binding, protein binding,	104	0.00433
GTPase activator activity,	104	0.00433
Extracellular matrix structural constituent,	101	0.0042
RNA binding,	89	0.0037
Ubiquitin-protein ligase activity, zinc ion binding,	89	0.0037
Serine-type endopeptidase inhibitor activity,	88	0.00366
Receptor activity, olfactory receptor activity,	85	.00354
Transcription factor activity, zinc ion binding,	81	0.00337
Transporter activity,	79	0.00329
Actin binding,	76	0.00316
Signal transducer activity,	76	0.00316
Nucleotide binding, protein serine/threonine kinase	75	0.00312
Nucleotide binding, ATP binding,	68	0.00283
Nucleotide binding, RNA binding,	66	0.00275
Transcription factor, sequence-specific DNA binding	63	0.00262
Receptor binding,	62	0.00258
Hydrolase activity,	60	0.0025
Nucleotide binding, RNA binding, protein binding,	59	0.00246
Protein binding, zinc ion binding,	55	0.00229
Structural constituent of cytoskeleton,	51	0.00212
Nucleic acid binding, zinc ion binding, metal ion binding,	50	0.00208
Molecular_function, protein binding,	48	0.002
RNA binding, protein binding,	48	0.002
Sugar binding,	48	0.002
Transcription factor activity, RNA polymerase II	46	0.00191
DNA binding, protein binding, zinc ion binding,	45	0.00187
Actin binding, actin binding, actin binding, structural	44	0.00183
Growth factor activity,	44	0.00183

For more complete information see Additional files.

**Table 3:** The distribution of biological processes in the blood protein SQL database

**Biological process**	**Count**	**Frequency**
Total	22069	1
Transcription, regulation of transcription, DNA-dependent,	811	0.03674
		
Regulation of transcription, DNA-dependent,	282	0.01278
Proteolysis,	278	0.0126
Transport,	250	0.01133
Translation,	244	0.01106
Signal transduction,	209	0.00947
Cell adhesion,	173	0.00784
Metabolic process,	164	0.00743
Protein amino acid phosphorylation,	153	0.00693
Protein ubiquitination,	139	0.0063
Ubiquitin cycle,	134	0.00607
Electron transport,	122	0.00553
Intracellular signaling cascade,	114	0.00517
Protein amino acid dephosphorylation,	105	0.00476
Multicellular organismal development,	101	0.00458
Phosphate transport,	98	0.00444
Protein folding,	90	0.00408
Cell adhesion, homophilic cell adhesion,	86	0.0039
Immune response,	85	0.00385
Microtubule-based movement,	85	0.00385
Signal transduction,G-protein coupled receptor protein	82	0.00372
Protein transport,	81	0.00367
mRNA processing, RNA splicing,	75	0.0034
Nucleosome assembly,	72	0.00326
Epidermis development,	61	0.00276
Cell adhesion, homophilic cell adhesion,	58	0.00263
Small GTPase mediated signal transduction,	57	0.00258
Cation transport, calcium ion transport,	56	0.00254
Cell surface receptor linked signal transduction,	56	0.00254
Cytoskeletal anchoring,	55	0.00249
Signal transduction, G-protein coupled receptor protein factor	55	0.00249
Signal transduction, G-protein coupled receptor protein	47	0.00213
Protein modification,	46	0.00208
Ion transport,	45	0.00204
Nucleosome assembly, chromosome organization	45	0.00204
Muscle contraction, cytoskeletal anchoring, development	44	0.00199
rRNA processing,	44	0.00199
Spermatogenesis,	44	0.00199
Carbohydrate metabolic process,	43	0.00195
Muscle development,	43	0.00195
Acute-phase response,	41	0.00186
Glycolysis,	41	0.00186
Intracellular protein transport,	41	0.00186

For further information see Additional files.

#### DNA binding factors and transcription factors

**Figure 8:**
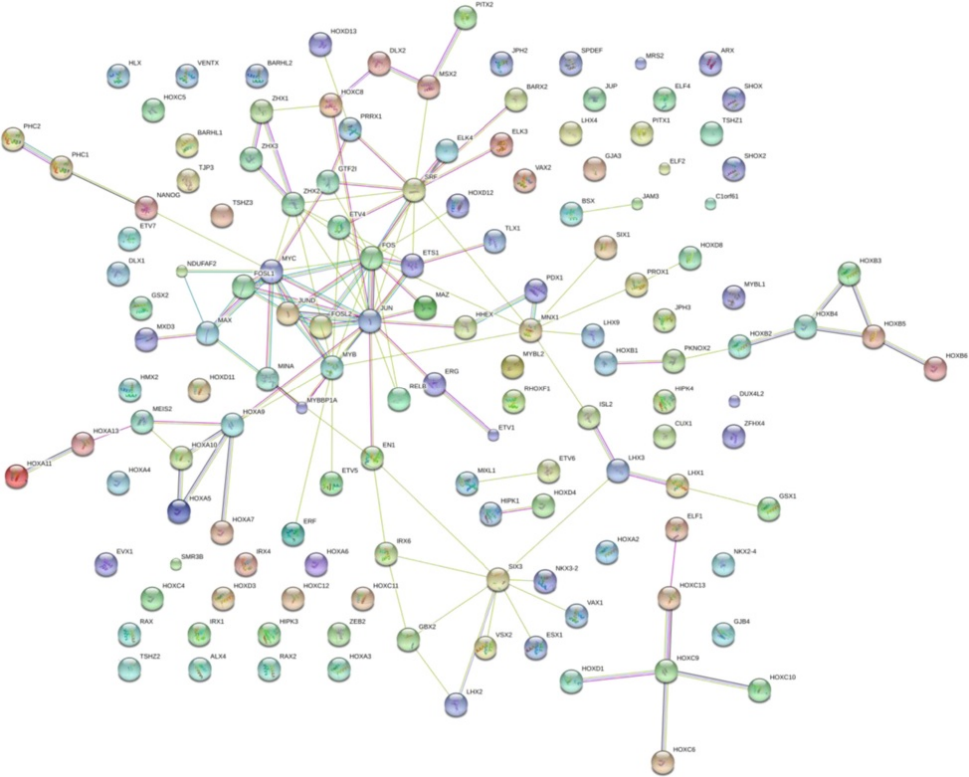
**The contents of the database queried for transcription-associated proteins are shown without filtering.** The full list of factors may be found in Additional file 1. The figure was produced using STRING evidence view. Colors: Green gene neighborhood; red gene fusion; blue concurrence; black co-expression; purple experiments; cyan databases; yellow text mining; and grey homology.

The database includes many DNA binding factors, DNA remodeling enzymes, RNA binding proteins, and receptor-associated signal pathway proteins. Zinc finger and other nucleic acid binding domains, including domains known to bind RNA have been detected in serum/plasma by a variety of methods [3,19] and predate the recent discovery of RNA and nucleic acids in blood [35]. The prevalence of proteins linked to transcriptional regulation, DNA remodeling, RNA binding and signal transduction was in agreement between groups and confirmed the unexpected presence of these circulating proteins [3]. Homeobox pathway proteins that are known to confer tissue and cell specificity were identified [36]. For example, the sine oculis homeobox homolog 3 (SIX3) was observed with many potential functional partners or related proteins. Members of the proto-oncogenes, or HLH transcription factor super family members, including myb, myc max and their associated binding factors, the AP-1 dimmers eg. isoforms of FOS, JUN, the serum response factor (SRF) and functionally associated proteins, such as ELK or ETS factors were detected . The transcription proteins in blood show a network relationship centered around FOS, JUN, ETS, MYB, MAX and HOX factors. In addition, many other transcription factors and proto-oncogenes containing DNA binding transcription factors, such as retinoblastoma-like 2 (RBL2) or V-Rel member of the Rel/NFKB family, were identified in serum/plasma with at least one peptide by mass spectrometry (Figure 8, Additional file 1).

#### Chromatin remodelling and nucleic acid modification enzymes

**Figure 9:**
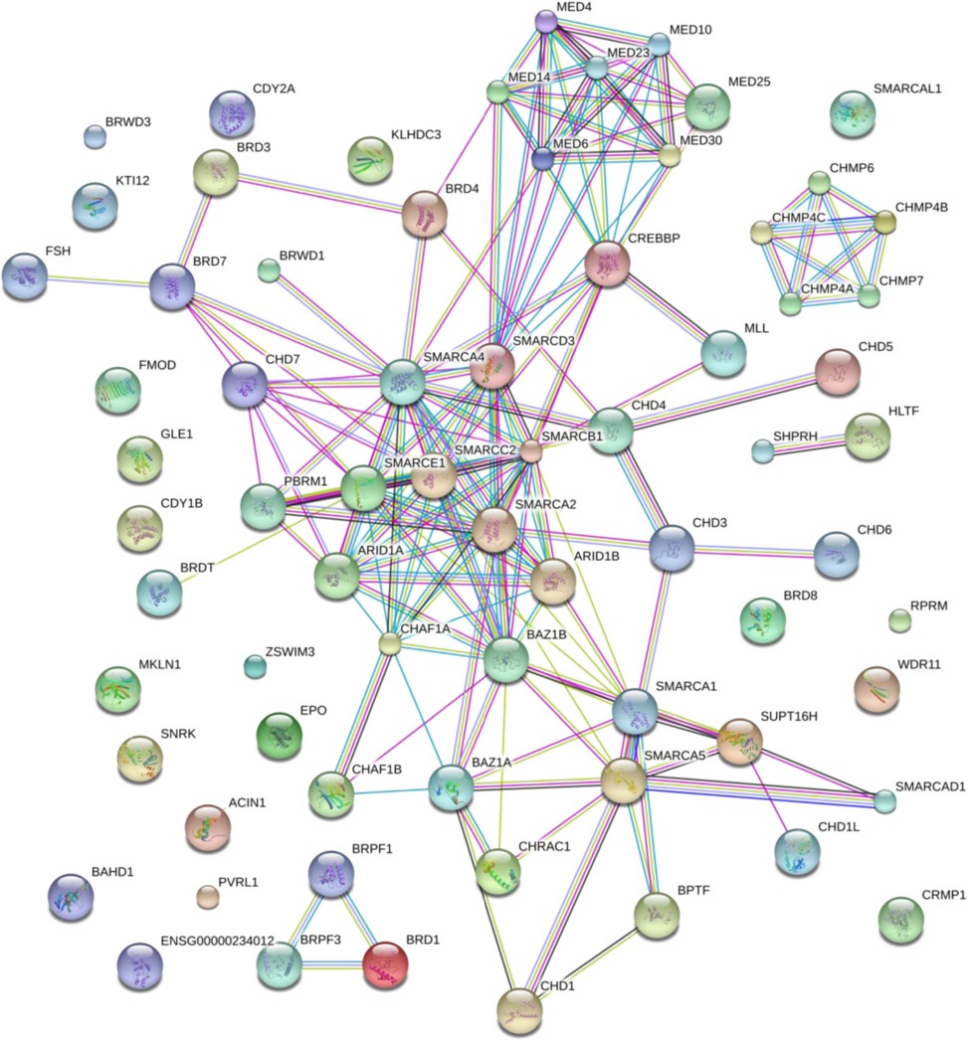
**DNA remodeling factors in human blood.** The contents of the database were queried for DNA remodeling-associated proteins and are shown without filtering. The full list of factors may be found in Additional file 2. The figure was produced using STRING evidence view. Colors: Green gene neighborhood; red gene fusion; blue concurrence; black co-expression; purple experiments; cyan databases; yellow text mining; and grey homology.

Proteins containing chromo and bromo domains, chromatin remodelling enzymes, histone modifications enzymes, as well as histones and high mobility proteins were clearly detected. The DNA remodeling proteins, including SMARC, bromo and chromo domains, SWI/SNF protein, including SMARC, bromo and chromo domains, SWI/SNF protein, histone modification enzymes, mediator proteins that are required for gene expression have been clearly detected with agreement by multiple peptides in serum/plasma. In addition, many other similar proteins exist in the FDBP of serum/plasma proteins: Actin and microtubule cytoskeleton proteins; myeloid/lymphoid or mixed-lineage leukemia (MLL) protein 3 homolog; chromosome assembly factors and the chromosome modifying proteins (CHMP); the E1A binding protein (EP); bromodomain and WD repeat-containing protein 1, required for the regulation of the cytoskeleton and cell shape [37]; mediator proteins (MED) that forge the physical connection between the DNA dependent transcription factors and the RNA polymerase; proteins with structural or functional similarity to transcription elongation factors (Figure 9, Additional file 2).

#### Zinc finger proteins and RNA proteins

**Figure 10:**
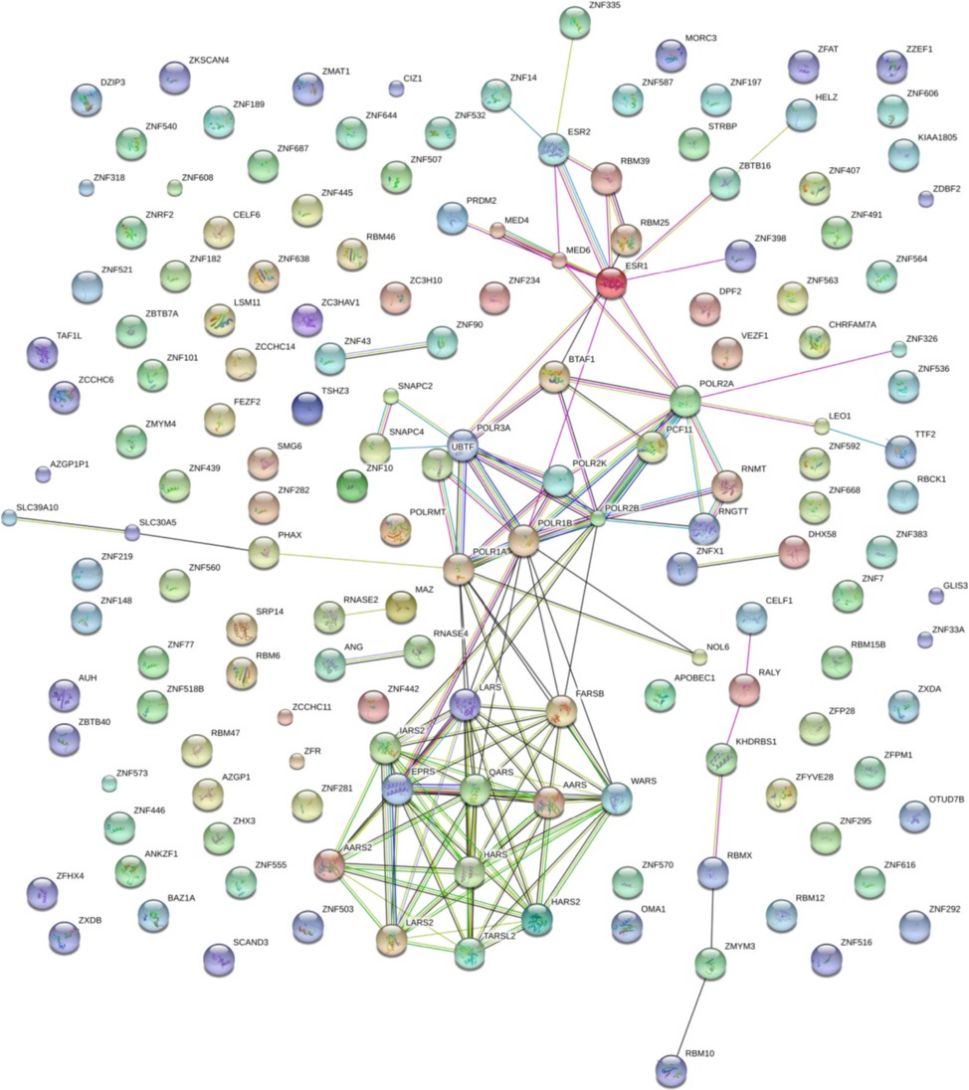
**RNA binding and zinc finger proteins.** The contents of the database were queried for RNA binding or zinc finger-associated proteins and are shown with filtering at n = 3. The full list of factors may be found in Additional file 3. The figure was produced using STRING evidence view. Colors: Green gene neighborhood; red gene fusion; blue concurrence; black co-expression; purple experiments; cyan databases; yellow text mining; and grey homology.

A large number of zinc finger proteins that have been shown to bind nucleic acid sequences were observed in serum/plasma. RNA/DNA binding regulatory factors that contain many different combinations of domains, including zinc, ring, PhD, SET, Scan, and many others, were abundant in the serum/plasma. As one of many examples, RNA-binding protein 39 (RBM39), also called hepatocellular carcinoma protein 1, is a transcriptional co-activator for steroid nuclear receptors ESR1/ER-alpha, ESR2/ER-beta, plus JUN/AP-1 and may be involved in splicing pre-mRNA [38] (Figure 10, Additional file 3).

#### SNAPS, SNARES, kinesin, secretion apparatus and exosome components

**Figure 11:**
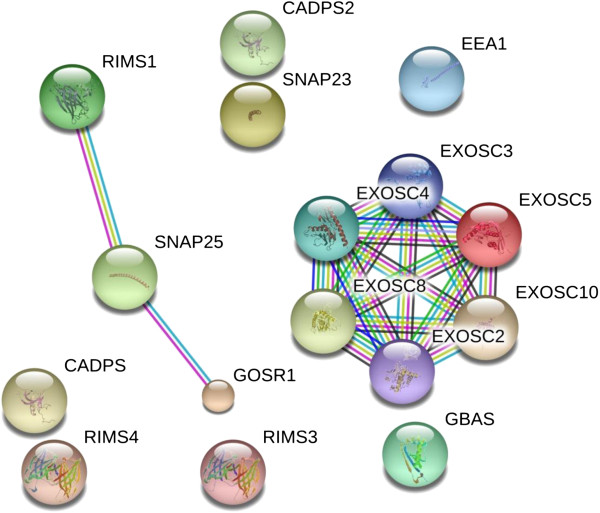
**The contents of the database were queried for secretion-associated proteins and are shown without filtering.** The full list of protein may be found in Additional file 4. The figure was produced using STRING evidence view. Colors: Green gene neighborhood; red gene fusion; blue concurrence; black co-expression; purple experiments; cyan databases; yellow text mining; and grey homology.

Many proteins that are functionally associated with the secretion pathway from cells or the formation of exosomes were detected in the serum or plasma. SNAP and SNARE proteins, such as GOSR1, that regulate synaptic membrane exocytosis via the membrane fusion of targeted vesicles were detected, such as GOSR1. Serum/plasma also contains many other similar proteins: The calcium dependent secretion activator CADPS and the regulator synaptic membrane exocytosis protein(s) RIMS4 were also detected. Endosome antigens, such as EEA1 were observed. Proteins associated with Golgi apparatus that governs secretion were also detected. Furthermore, and exosome components 8, 10 and RRP46, among others, have been observed (Figure 11, Additional file 4).

#### Cellular receptors and signal transduction factors

**Figure 12:**
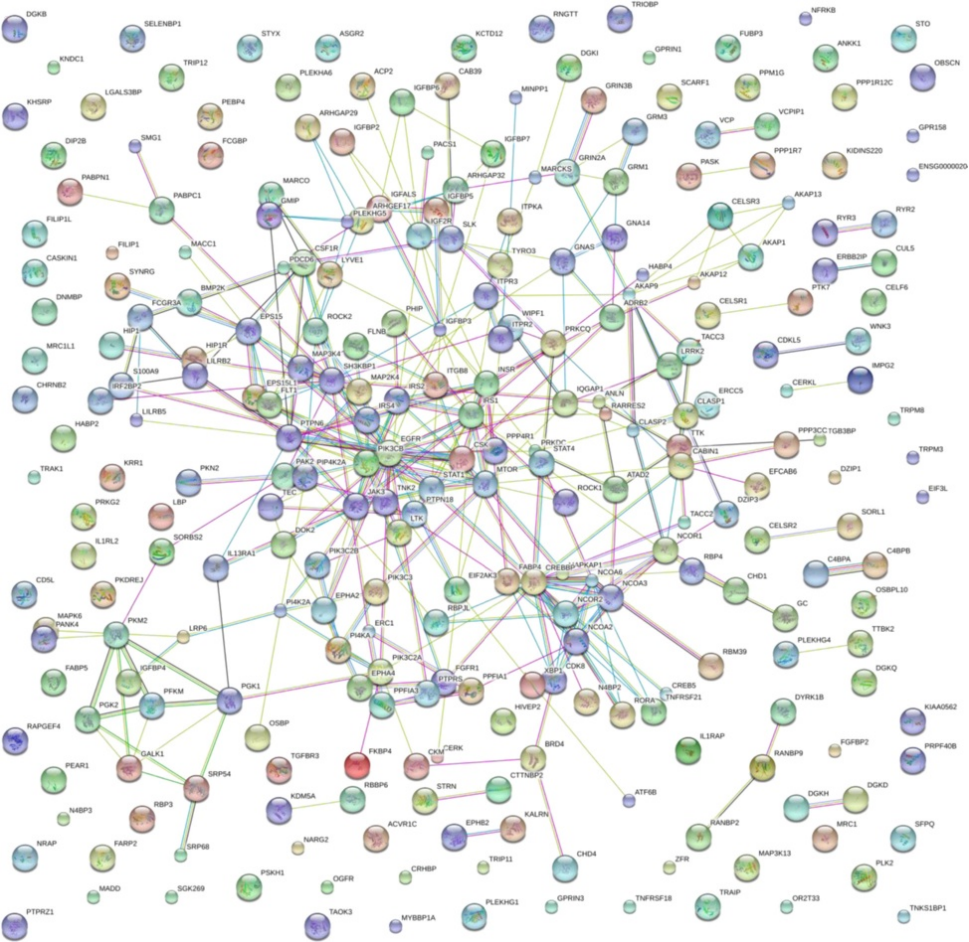
**The receptor and signal transduction proteins in human blood serum or plasma.** The contents of the database wee queried for receptors, kinases, phosphatase and cell signalling-associated proteins and are shown with filtering at n = 5. The full list of factors may be found in Additional file 5. The figure was produced using STRING evidence view. Colors: Green gene neighborhood; red gene fusion; blue concurrence; black co-expression; purple experiments; cyan databases; yellow text mining; and grey homology.

An extraordinary wealth of ligands, receptors, receptor-associated-kinases, phosphatases, adaptor proteins, nucleotide-binding G proteins, exchange factors, activating factors and many other binding proteins along the signal transduction pathways of cells have now been clearly observed in plasma. For example the protein kalirin (KALRN) that apparently is a RHOGEF kinase isoform was clearly detected. Well-known receptors, such as the adrenergic (ADRA) or GABA receptor (GABBR), plus unknown or unusual receptor factors were detected. For example, the BTB/POZ domain-containing protein KCTD12, which may be a subunit of the GABA-B receptor, was observed. Thyroid receptor-interacting protein 11, encoded by the TRIP11 gene, functionally interacts with thyroid hormone receptors alpha and beta, and the Retinoblastoma (RBB) protein [39]. Receptors with roles that mediate specific cell surface recognition events such as immune response, cell growth, differentiation, metabolism and survival, including Fc, MHC, scavenger or Cadherin and EGF LAG seven-pass G Protein Coupled Receptors (GPCRs) -type 3, Integrin beta-8 (ITGB8) were detected. The receptor-type tyrosine-protein phosphatase beta that is a negative regulator of growth factor and cadherin receptors was also identified. There were members of both trimeric and monomeric G-protein pathways, such as small G protein signalling modulator 3, or the metabotropic glutamate receptor 3 which signals via a timeric GTPase (G protein) that inhibits adenylate cyclase activity. Rho guanine nucleotide exchange factor 17 (ARHGEF17) is a protein that swaps GDP for GTP to enable the function of monomeric GTPase (G protein). In contrast, Rho GTPase-activating protein 29 (ARHGAP29) is a protein that causes the catalysis of GTP to GDP, turning the G- protein switch off. Ryanodine receptor 2 (RYR2) is found in cardiac muscle as part of a calcium channel and mutations in RYR2 gene may be associated with Alzheimer’s, tachycardia, arrhythmogenic right ventricular dysplasia [40]. Inositol 1,4,5-trisphosphate receptor type (IPTR3) was observed at least 25 times and may play a role in a variety of signalling networks. Insulin receptor substrate 4 may act as a second messenger that participates in the flow of information via a chain of phosphorylation events. BRCA1-associated RING domain protein 1 is a probable E3 ubiquitin-protein ligase that mediates the formation of ‘Lys-6’-linked polyubiquitin chains in DNA damage repair, ubiquitination and transcriptional regulation. In addition, a probable ubiquitin carboxyl-terminal hydrolase was also observed. A great number of well-known cellular signalling proteins, including kinases and phosphatases were detected by multiple groups in agreement. It is striking to consider the many types of proteins that are now known to mediate signalling within cells and were apparently found in the plasma at detectable concentrations (Figure 12, Additional file 5).

#### Cytokines, chemokines, interleukins and tumor necrosis factor receptors and binding proteins

**Figure 13:**
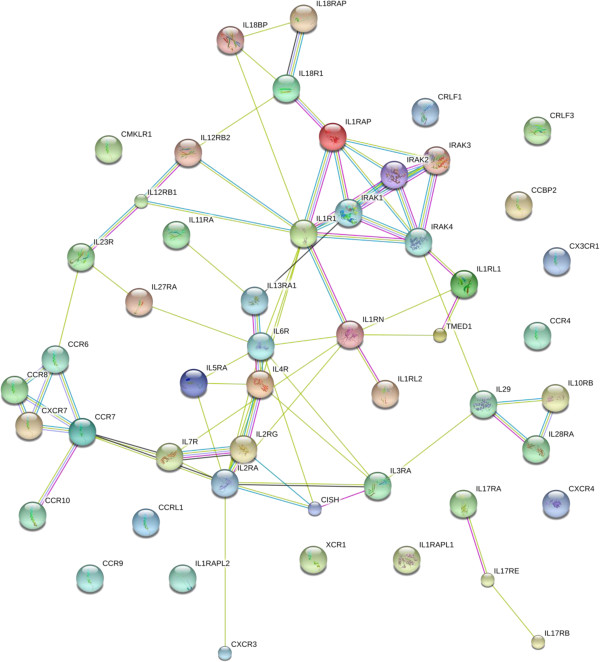
**The cytokine, chemokine and interleukin proteins of human blood plasma or serum.** The contents of the database were queried for cytokines, chemokines, interleukins and tumor necrosis factor associated proteins and are shown without filtering. The full list of factors may be found in Additional file 6. The figure was produced using STRING evidence view. Colors: Green gene neighborhood; red gene fusion; blue concurrence; black co-expression; purple experiments; cyan databases; yellow text mining; and grey homology.

The chemokines, cytokines, interleukins, and tumor necrosis factor ligands were apparently below the detection limit of the sample preparation and electropsray LC-MS systems employed [10,41-44]. However, the receptors and associated proteins of chemokines, cytokines, tumor necrosis factors and interleukins were observed. For example TNF receptor-associated protein 1 (TRAP1) was clearly detected. Interleukin 1 receptor-like 2 (IR1RL2) was well detected from plasma. Receptors for proteins with homology to TNFalpha and other cytokines that function in tissue differentiation were identified in serum/plasma, such as the receptor tyrosine kinase (RET) that binds the Glial cell line-derived neurotrophic factor. A wide range of proteins annotated as part of cytokines, chemokines and interleukins pathways were observed (Figure 13, Additional file 6). While cytokine and chemokine receptors and binding proteins were detected, the ligand factors were below detectable levels, as expected.

#### Growth factor receptors and binding proteins

**Figure 14:**
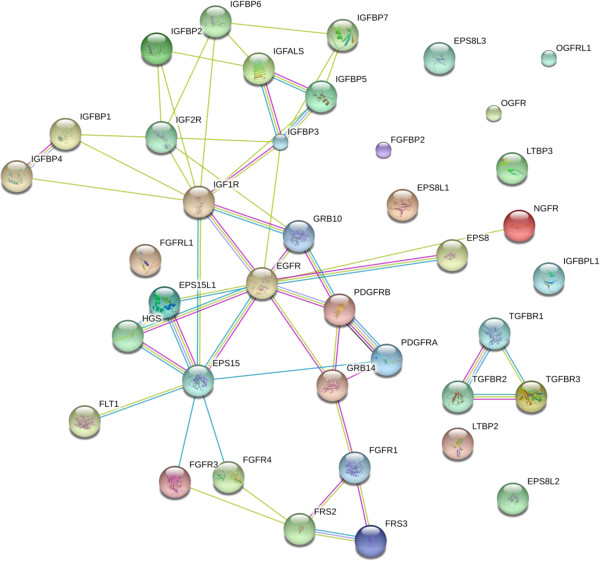
**The growth factor proteins of human blood plasma or serum.** The contents of the database were queried for growth factor associated proteins and are shown without filtering. The full list of factors may be found in Additional file 7. The figure was produced using STRING evidence view. Colors: Green gene neighborhood; red gene fusion; blue concurrence; black co-expression; purple experiments; cyan databases; yellow text mining; and grey homology.

Epidermal growth factor receptor (EGFR) and its substrates were observed in plasma with multiple peptides. Ligands that are present in higher concentrations than cytokines, such as Insulin like growth factor [41] were well observed by multiple groups. Insulin-like growth factor-binding receptor that contains an IGFBP N-terminal and a Thyroglobulin type-1 domain was detected in the plasma; the ligand insulin like growth factor has previously been identified in blood [41]. Proteins with homology to growth factors, such as the multiple epidermal growth factor-like domains 8, which is an EGF domain protein involved in signalling, were detected. Receptor tyrosine-kinase Erb interacting and binding proteins were observed. In addition, fibroblast growth factor, opioid, and FMS like tyrosine kinase growth factor receptors or binding proteins were observed (Figure 14, Additional file 7). However, while growth factor receptors and binding proteins were detected, the growth factors themselves, that exist at much lower concentrations in blood, were below detection limits.

## Discussion

### FDBP strategy

A number of strategies have been suggested for the analysis of proteomic data that require proteomic-specific data storage and analysis platforms. The data analysis strategies generally advocate the storage of raw data in xml or text files with proteomics-specific software routines to manage, analyze and summarize the data [45-47]. In contrast, we proposed that the generic data analysis systems such as SQL, BLAST, and a generic statistical analysis system (SAS) may be used to organize and analyze proteomic data, using this broadly available and well tested software [10,12,20,23,29,48]. When considering the choice of a data analysis system, it is important to note the differences in proteomic versus genomic data. Genomic data is the linear character sequence of about 3 billion base pairs. The proteome corresponds to only about 1% of the genome, comprising 22,000 protein types to date. In addition, there is a 3 to 1 compression of the data from bases to amino acids and so the protein sequence data is no more than 0.3% that of the genome. In many instances, only a few representative peptides have been recorded from each protein, and so the sequence data collapses to less than 0.1% of the genome sequence. However, individual peptides may be detected repetitively and these detections can be stored as numeric information. Hence proteomics data sets will contain at least a thousand fold less sequence information than genomic databases but have much more numerical data including m/z values and continuous intensity values from the parent and fragment ions [10,11]. The large amount of continuous fragment m/z and intensity data must be connected to the relatively small amount of protein and peptide sequences or masses [M+H], that are ordinal or nominal variables, in order to compute the differences in intensity values over treatments [10,12,20,23,29,48]. The ion intensity data must be linked to the protein, peptide, and m/z information in a format that will permit immediate statistical analysis by generic routines [10-12].

### Analytical error in protein identification

When a highly purified protein is analyzed by LC-MS/MS it is sometimes possible to achieve complete sequence coverage and therefore unambiguous identification between highly related sequences. However, when many proteins are identified and quantified simultaneously, the peptide coverage of each protein is not complete and so there may be more than one protein sequence that matches the detected peptides. In some cases, where only a few peptides are detected there may be no way to rule out related proteins without subsequent investigation. Most proteomic scientists support the concept of creating large databases of proteins from different sources, but there are no universally accepted processes for creating such databases. We have chosen to collect data on serum/plasma proteins from several published sources to create a FDBP that depends on the veracity of the methods used to collect, combine and analyze the data to avoid the pitfalls that might spuriously incorporate inappropriate molecules into the FDBP. The proteins of human blood have been separated by various methods, including a variety of chromatographic strategies for separation prior to ionization and the MS/MS spectra were collected with commercially available quadrupole or ion trap instruments [23,29]. Together these methods yield a large number of peptides correlated to a small number of proteins in sharp contrast to random expectation. It’s has been suggested that three peptides many be a reasonable standard to limit false positive rates into protein databases based on random protein libraries and calculations based on random spectra or noise spectra also indicate that the false positive rate (type I error) estimated from peptide-to-protein distributions was in agreement with independent goodness of fit tests of the protein spectra and graphical approaches [25,27,28].

### BLAST versus SQL

Less than half of the human blood proteins have closely related homologs in the FDBP and there may be partial sequences, splice variants, or allelic forms of proteins in the FDBP that differ by at least one amino acid. In some instances, it may be desirable to collapse proteomic data to a set of unique or representative protein sequences. It has been previously shown that the protein databases can be collapsed into a smaller number of protein types by listing all perfect subsets under the longest representative sequence, or collapsing the proteins using BLAST analysis with similar results [10-12,20,23,29]. The BLAST criteria of 75% full length homology and 20 contiguous amino acids [20,23,29] is a standard method to assign structural identity to different proteins. The standard BLAST algorithm can be used to quantify the extent of identity between proteins and domains and to annotate them. We conclude the BLAST algorithm is a sufficient metric to distinguish protein types that flexibly discovers relationships between proteins which can then be directly captured in an SQL database. The strategy of parsing control or test data sets, using existing software, such as SQL, for assembly prior to BLAST or statistical analysis by generic routines like SAS or R, may be appropriate for annotation and quantitative analysis of proteomic data. Moreover, as shown here, the results of BLAST can be conveniently stored in the same SQL source database, dramatically simplifying the organization and increasing archival quality of the data with convenient analysis and graphical presentation the generic statistical analysis software such as SAS. We conclude that up to 17,506 protein types might exist in blood based on homology to proteins where peptides were obtained and of these about 7,707 of these are identified with reasonable certainty. The redundant protein count is the number of times any MS/MS spectrum has been correlated to a peptide from any protein sequence(s). The same peptide, and therefore protein(s), may be detected in replicate experiments and the redundant peptide count provided yields an estimate of the relative levels of detection. Some peptides are found in protein sequences that are identical between protein libraries, and the many equivalent library accession numbers may be concatenated with semi colons, for convenience without losing information. Multiple protein sequences that are exactly the same can be eliminated by SQL with a simple automated function to yield a distinct protein list of all implicated proteins that differ by at least 1 amino in the protein sequence. Hence the redundant versus distinct peptide and protein counts of 10,138 distinct proteins with 3 peptides are convenient and easily reproducible metrics of the relative levels of detection and the number of potential proteins using commonly available software. Considered together, the direct comparison of BLAST versus SQL indicate that about 70% of the proteins detected in blood by three peptides or more have no other close homologues in circulation while an minority of proteins may have other similar protein variants, isoforms or related sequences in circulation.

### Unique or characteristic peptide sequence analysis

Some fourteen thousand of the reported serum/plasma proteins map to only one distinct protein sequence that cannot be related to any other protein by BLAST but these proteins can still be summarized at the peptide and protein level using SQL. Moreover it is important to remember that mass spectrometers most typically detect peptides and not proteins. Thus a summary on the basis of unique peptides that can be unambiguously analyzed by LC-ESI-MS/MS is a meaningful metric for mass spectrometry experiments. If we accept the set of proteins detected by at least one unique or characteristic peptide not found in any other protein, as list of 12,130 proteins are apparently in the blood and from these a conservative estimate of 3,858 proteins in the blood with reasonable certainty was obtained.

### Biological sources of error

It seems unlikely that cellular proteins observed with three or more peptides, and in agreement between different research groups, could be identified erroneously. However, it remains possible that at least some of these proteins could be released from cells during blood collection or processing. Some of the observed blood proteins may have been released from the site of wounding and diffused into the blood from the damaged skin tissue or cells. The activation and degranulation of blood cells is known to sometimes occur during the formation of serum and might release the contents from cells that burst during blood clotting. Red blood cells are anucleate and so they might not seem like a rich source of nuclear factors. Similarly, platelets are anucleate and so at least superficially [49] they are unlikely source of DNA remodeling enzymes and transcription factors. Direct measurements of secreted platelet proteins by LC-MS make little mention of such cellular factors except for well-known secreted proteins such as 14-3-3 proteins and a single PI3K isoform and a few other similar proteins [50,51]. It is known that neutrophils and potentially other blood cells use expelled DNA as a net or snare to entrap bacteria [52]. It remains possible that white blood cell degranulation during processing results in expulsion of nucleic acids and their binding proteins.

Analysis of the proteins released from leukocytes was used to rule out the degranulation of white blood cells during collection as the source of the transcription factors and other nuclear proteins in the blood. We tested the hypothesis that the observed transcription factors, receptors, signaling enzyme, DNA remodeling and other signaling proteins observed in the FDBP were merely secreted by white blood cells during degranulation. To test whether DNA binding factors and other cellular proteins were released from white cells, human neutrophils were isolated and degranulation was stimulated with the combination of cytochalasen B and the bacterial peptide fMLP. The results of the neutrophil stimulation experiment showed that very few of the observed cellular factors in blood were secreted from these abundant white blood cells during degranualtion (not shown). The abundance of cellular and nuclear materials in plasma samples seems to indicate that a very efficient system for releasing proteins from cells, such as secretion or the release of exosomes, must be present to account for such a large concentration of so many proteins [7,8,35,53,54].

### Utility of the federated database of blood proteins

The FDBP will be useful only if the data are reliable and easy to search or to manipulate. The above paragraphs give the reasons for believing that highly reliable data may be derived from the FDBP. To make the FDBP easily useful, we placed all of the data in a SQL database to permit analysis of the data. The generic SQL and SAS system can also be used to capture, organize and analyze the results of bioinformatic algorithms such as BLAST or the results of GO term analysis, as shown here. The FDBP contains the BLAST and GO term data for the proteins listed that can be rapidly and conveniently summarized by a generic statistical analysis system such as R or SAS [10]. The results of the many additional calculations are also made available in the provided excerpts of SQL databases where the data may be analyzed and graphically presented with SAS. The generic data systems SQL and SAS are sufficient to analyze proteomics data and can derive the necessary attributes and distributions of the data.

A further capacity to provide the calculated parent and fragment m/z values for the peptides in the FDBP is a significant advantage in designing experiments for unambiguous identification and quantification by precise mass spectrometric methods [10-12]. The mapping of the peptides to the different protein sequences in the FBPD will help to interpret proteomic results and for the planning of experiments to make unambiguous protein determinations. Comparing the attributes between the different related sequences or subsequences may be informative and so collapsing the data into one representative protein from each protein type may result in the loss of valuable information. Where a feature of interest is discovered in the data that span several similar, but distinct protein sequences, it is a simple task to determine if the data available support the presence of one or more related proteins, and which peptides are unique to each protein, on a case by case basis in SQL so long as all data is made available. A separate intensity or frequency calculation can be made for each different protein sequences regardless of homology to other proteins [10-12,20,23,29]. Where such discrimination between partial sequences, splice variants, predicted proteins or allelic forms is made by subsequent experiments, it will first be required to compare all of the protein sequences together in the same database to look for sequences unique to specific proteins.

#### Sensitivity

The limit of quantification of an LC-ESI-MS/MS experiment for a pure compound is typically about 100 femto mol to 1 pico mol injected on column. Testing purified protein digests on an LC-ESI-MS/MS running at 2 μl per minute via an electropsray into an ion trap showed 10 f mol of standard proteins may be reproducibly and confidently identified, 1 femto of peptide on column seems to be at the detection limit and 100 atto mol of digest on column was typically beyond the sensitivity of a simple LC-ESI-MS/MS method for automatic identification [19,55]. Based on the above estimates of system sensitivity, we can calculate the range of required concentrations of the above mentioned regulatory proteins in order for them to be detected in the approximate volume of serum/plasma used in the LC-MS experiments summarized here. Since the plasma proteins were apparently detectable by LC-ESI-MSMS then there must be at least 1 to 10 femto mol of the serum/plasma peptide on the column for identification by a simple ion trap. Anderson and Anderson [56] estimated that the concentration of proteins that leak from tissue and diffuse from cells could reach the nanogram per ml of blood. A protein with a mass of 50,000 Da present at 1 ng per ml has a concentration of about 20 pico molar. Therefore, in order to detect a protein in the 1 ng per ml range in blood, a starting sample in the tens to hundreds of microlitres of blood would have to be efficiently captured and fractionated, to deliver 1-10 femto mol in a single discrete fraction within detection limits and in agreement with the sample sizes used in some of the studies cited here. These calculations are consistent with previous observations of proteins known to be at least as low as 1 ng/ml which have been observed by mass spectrometry from a sample volume in the order of tens to hundreds of microliters [19,55]. From these calculations, we infer proteins in the ng/ml or roughly pico molar range are near the limit of robust detection by electrospray with a simple ion trap in an unbiased LC-MS experiment after a simple chromatographic pre-fractionation of small samples [19] and this estimate has been confirmed [43]. Protein biomarkers known to be in the range of 1 ng/ml such as thyroglobulin and others have been repeatedly detected by mass spectrometry [19,55].

##### Cellular proteins in serum/plasma

Tissue or cell leakage [56], secretion [11] or release of membrane-bound exosomes [35] have been proposed as the pathways by which cellular proteins, such as nucleic acid binding proteins, might reach the plasma. It now appears that there are significant amounts of intact nucleic acid strings in plasma and that enough fetal DNA is released into the blood stream of a pregnant mother to provide a draft fetal genome sequence [57]. The existence of nucleic acid polymers in plasma probably leads to the presence of their binding proteins in circulation. Nucleic acid binding proteins such as histones and high mobility group proteins have previously been detected in serum/plasma at concentrations as high as 1 to 40 ng/ml, using Western blot and ELISA [58-62]. The cytokine receptors or growth factor receptors that are known to exist in serum/plasma in the ng per ml range were detected by LC-MS. In contrast, there were no detections of cytokines or growth factors, that exist at pico gram per ml levels: From these observations, we conclude that the concentrations of the proteins effectively detected in serum/plasma by mass spectrometry closely match the established detection limits of the LC-MS systems referred to in this review [10,11,19,42-44]. The presence of many serum/plasma proteins, associated with circulation or transport functions, proteolysis and metabolic processes agrees with traditional views of the circulating proteins [5,6]. However our meta analysis showed that nuclear proteins with a role in DNA transcription and/or RNA binding or metabolism as well as proteins associated with signal transduction from the plasmalemma receptor pathways were identified with impressive agreement between different groups, confirming previous reports [3,19,41,63,64].

##### Biomarker potential

The collection of protein and peptide information, along with the proteins’ cellular locations and molecular functions, together with expression patterns in differentiated tissues and cells provides a powerful means for elaborating hypotheses about potential biomarkers in serum/plasma, before validating them by targeted assays. The detection of zinc finger and other nucleic acid binding domains known to bind RNA by mass spectrometry [3,19] correlates with the recent discovery of circulating RNA in blood [35]. Purified exosomes from blood may also contain nucleic acids and their binding proteins [35]. It may be possible to identify and quantify the presumably non-coding RNA (ncRNA) in the plasma that will help shed light on the function of the RNA binding proteins detected in blood by mass spectrometry. Complexes of nucleic acids and proteins, including histones, are estimated to circulate at the level of several hundreds of nanograms per ml [62]. It has been suggested that modified histones, complexed with nucleic acids in the plasma, may be biomarkers of cancer [59,60]. High mobility group proteins and histones may be secreted by cells in response to immunological activation and have been reported to be a biomarker of lupus or other diseases reaching concentrations as high as 40 ng/ml in blood [58-60]. If relatively non-specific DNA binding proteins such as HMG or histones may serve as biomarkers, other more specific nucleic acid binding proteins might also have some clinical significance. However, for any plasma protein to serve as a reliable biomarker, it is important that the blood collection and pre-anaytical procedures be standardized and documented [65].

#### Towards the definitive analysis of blood proteins

The reliability of the data presented here was previously established using a variety of statistical methods including distribution between NP versus XP protein libraries, agreement between the data sets, peptide to protein distributions, and non-random distribution of the data over specific GO term categories in agreement with expectation values from goodness of fit tests of the MS/MS spectra [20,23,29]. The peptide to protein distribution of the database *in toto* is consistent with the veracity of the correlation algorithms used by the different research groups [23,29]. To date it seems that LC-ESI-MS/MS of serum/plasma has revealed a total of 12,130 proteins detected with at least 1 unique or characteristic peptide not found in any other sequence and other these 3858 showed reasonable certainty. In contrast, 7,707 high confidence blood proteins were calculated by BLAST. The related protein sequences can be analyzed using SQL or BLAST, but this additional level of collapse is not necessarily required to make comparisons of detection frequency by Chi square or mean ion intensity by ANOVA to detect proteins of potential interest. At present, routine monitoring of proteins in blood requires the use of monoclonal antibodies for standard ELISA assays. However, there are not sufficient immunological reagents to confirm the majority of the blood proteins discovered to date by mass spectrometry. The limit of detection of mass spectrometry may rival that of ELISA after reproducible partition chromatography [19] and this level of sensitivity has been confirmed [43]. Many blood proteins discovered by mass spectrometry using sensitive ion traps are near or below the present quantification limits of ELISA or LC-ESI-MS/MS for routine analysis. Extensive experimentation based on affinity reagents and/or mass spectrometry will be required to establish the protein or peptide biomarkers of blood with the acceptable standard of certainty provided by three independent biophysical or biochemical methods in agreement.

## Conclusion

**Figure 15:**
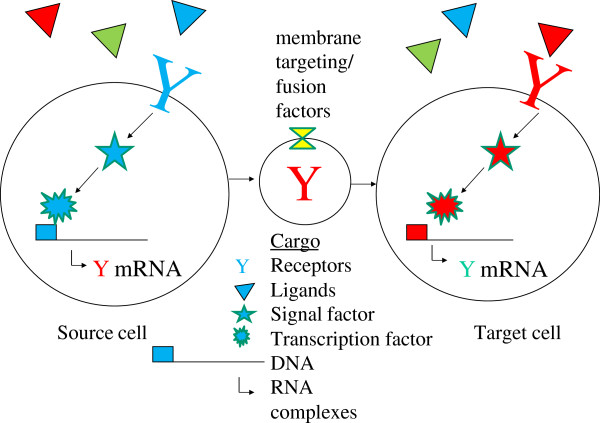
Note that exosomes may contain proteins like ligands, receptors, transcription factors or RNA and potentially DNA that may alter the target cells fate, differentiation or functions.

In conclusion, serum/plasma contains an abundance of nuclear proteins including transcription factors, DNA remodeling enzymes, RNA binding proteins, cellular receptors and their associated proteins, or enzymes that might serve as biomarkers of cell and tissue specific diseases. These unexpected cellular factors indicate that the blood hosts many cellular proteins, including nucleic acid binding proteins and factors associated with intercellular signaling. The nucleic acid binding and signaling factors in serum/plasma may still be functional. The transport of secreted signal factors to distal cells may have peri- or endocrine functions. The presence of so much genomic DNA, RNA and their binding proteins in circulation, presumably including mobile genetic elements [66], cannot preclude the transfer of transformative agents between cells (Figure 15). In conclusion, our work confirms the reliable detection of transcription factors, chromatin remodeling factors, nucleic acid binding proteins and receptor-mediated signaling enzymes in blood fluid across different proteomic studies. It has been suggested that cancerous cells or stem cells may secrete exosomes that contain receptors or other factors such as RNA or transcription activators that if expressed in target cells could permanently affect the recipient cells’ fate or differentiation. A set of cellular regulatory proteins that show great promise as biomarkers and biotherapeutic proteins have been detected with high confidence in human serum and plasma.

## Competing interests

The authors declare that they have no competing interests.

## Authors' contributions

JGM performed the STRING analysis; PB performed the SQL analysis and wrote the parsers; JCS, conceived the project and supervised the writing of the project proposal; FB supervised the laboratory experiments; JGM, FB, and JCS wrote and edited the manuscript. All authors read and approved the final manuscript.

## Supplementary Material

Additional file 1 **The contents of the database queried for transcription-associated proteins are shown without filtering.** The full list of factors may be found in Additional file 1. The figure was produced using STRING evidence view. Colors: Green gene neighborhood; red gene fusion; blue concurrence; black co-expression; purple experiments; cyan databases; yellow text mining; and grey homology. Click here for file

Additional file 2 **DNA remodeling factors in human blood.** The contents of the database were queried for DNA remodeling-associated proteins and are shown without filtering. The full list of factors may be found in Additional file 2. The figure was produced using STRING evidence view. Colors: Green gene neighborhood; red gene fusion; blue concurrence; black co-expression; purple experiments; cyan databases; yellow text mining; and grey homology. Click here for file

Additional file 3 **RNA binding and zinc finger proteins.** The contents of the database were queried for RNA binding or zinc finger-associated proteins and are shown with filtering at n = 3. The full list of factors may be found in Additional file 3. The figure was produced using STRING evidence view. Colors: Green gene neighborhood; red gene fusion; blue concurrence; black co-expression; purple experiments; cyan databases; yellow text mining; and grey homology. Click here for file

Additional file 4 **The contents of the database were queried for secretion-associated proteins and are shown without filtering.** The full list of protein may be found in Additional file 4. The figure was produced using STRING evidence view. Colors: Green gene neighborhood; red gene fusion; blue concurrence; black co-expression; purple experiments; cyan databases; yellow text mining; and grey homology. Click here for file

Additional file 5 **The receptor and signal transduction proteins in human blood serum or plasma.** The contents of the database wee queried for receptors, kinases, phosphatase and cell signalling-associated proteins and are shown with filtering at n = 5. The full list of factors may be found in Additional file 5. The figure was produced using STRING evidence view. Colors: Green gene neighborhood; red gene fusion; blue concurrence; black co-expression; purple experiments; cyan databases; yellow text mining; and grey homology. Click here for file

Additional file 6 **The cytokine, chemokine and interleukin proteins of human blood plasma or serum.** The contents of the database were queried for cytokines, chemokines, interleukins and tumor necrosis factor associated proteins and are shown without filtering. The full list of factors may be found in Additional file 6. The figure was produced using STRING evidence view. Colors: Green gene neighborhood; red gene fusion; blue concurrence; black co-expression; purple experiments; cyan databases; yellow text mining; and grey homology. Click here for file

Additional file 7 **The growth factor proteins of human blood plasma or serum.** The contents of the database were queried for growth factor associated proteins and are shown without filtering. The full list of factors may be found in Additional file 7. The figure was produced using STRING evidence view. Colors: Green gene neighborhood; red gene fusion; blue concurrence; black co-expression; purple experiments; cyan databases; yellow text mining; and grey homology. Click here for file
